# Mapping the clinical landscape of multifunctional CAR T cells: Targets, trends, and synergies

**DOI:** 10.1016/j.ymthe.2026.02.006

**Published:** 2026-02-06

**Authors:** Isabella Elias Yonezawa Ogusuku, Nele Knelangen, Boris Engels, Marion Subklewe, Dominik Lock

**Affiliations:** 1 Miltenyi Biotec B.V. & Co. KG, Bergisch Gladbach, Germany; 2 Department of Medicine III, University Hospital, LMU Munich, 81377 Munich, Germany; 3 Laboratory for Translational Cancer Immunology, Gene Center, LMU Munich, 81377 Munich, Germany; 4 German Cancer Consortium (DKTK), Partner Site Munich, 81377 Munich, Germany

**Keywords:** chimeric antigen receptor, CAR T cell, multifunctional, next-generation, combination

## Abstract

Chimeric antigen receptor (CAR) T cell therapies have achieved remarkable and durable clinical responses in several hematological malignancies, yet their broader impact remains limited by toxicities and reduced efficacy, especially in solid tumors. Building on the successes and limitations of approved CAR therapies, more than 1,800 clinical trials were registered over the past decade. As of September 2025, 33% investigate next-generation CAR T products that incorporate auxiliary features intended to enhance safety and therapeutic efficacy (multifunctional CAR T cells). In this systematic review, we screened such multifunctional approaches across the complete landscape of CAR T cell trials registered at Clinicaltrials.gov. To provide a comprehensive discussion of this emerging paradigm and better understand its real-world impact, we classified multifunctional CAR trials as either safety or efficacy enhancements and monitored their registration frequency over the past 20 years. We describe each type of multifunctional CAR T cell currently present in clinical trials and discuss their subtypes and pervasiveness alongside the clinical results thus far available. Taken together, our mapping highlights three main developments: efficacy enhancements have advanced more rapidly than safety strategies both in number and diversity; multitargeting approaches have gained momentum; and combined strategies that simultaneously incorporate safety and efficacy enhancements are emerging to bridge individual shortcomings.

## Graphical abstract

**Figure:**
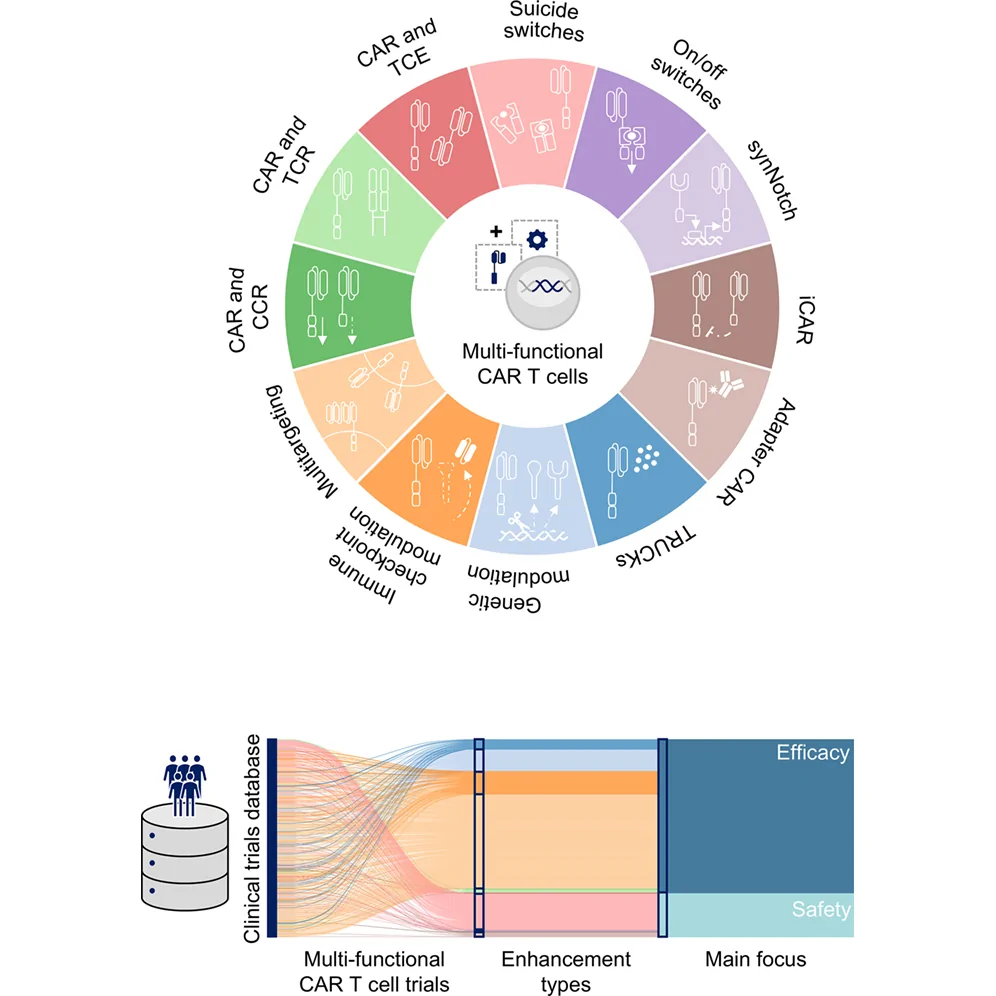

## Teaser

Ogusuku, Knelangen, and colleagues review and map next-generation, multifunctional CAR T cell strategies across all registered studies in Clinicaltrials.gov, highlighting the rise of multiantigen targeting, major trends in safety and efficacy enhancements, and emerging approaches that integrate both.

## Introduction

Chimeric antigen receptor (CAR) T cells have revolutionized cancer therapy. Their first US Food and Drug Administration (FDA) approval in 2017 marks both the first cell therapy for cancer and the first gene therapy in the United States. Since then, CAR T cells have received six more FDA approvals for the treatment of hematological malignancies (Table 1) and remain at the forefront of cancer immunotherapy as next-generation designs advance in the clinic.

**Table 1:** FDA-approved CAR T cell therapies

Generic name	Brand name	Indication	Target antigen	Targeting domain	Hinge-transmembrane-signaling domains	Gene transfer	Cell source	Reference
Tisagenlecleucel	Kymriah	diffuse large B cell lymphoma	CD19	mouse FMC63	CD8a-CD8a-41BB-CD3ζ	SIN lentivirus	T cells	O’Leary et al.^1^
		B cell acute lymphoblastic leukemia						
		follicular lymphoma						
Axicabtagene ciloleucel	Yescarta	follicular lymphoma	CD19	mouse FMC63	CD28-CD28-CD28-CD3ζ	γ-retrovirus	T cells	Sharma et al.^2^; Bouchkouj et al.^3^
		large B cell lymphoma						
Brexucabtagene autoleucel	Tecartus	B cell acute lymphoblastic leukemia	CD19	mouse FMC63	CD28-CD28-CD28-CD3ζ	γ-retrovirus	T cells	Deshpande et al,^4^; Bouchkouj et al.^5^
		mantle cell lymphoma						
Lisocabtagene maraleucel	Breyanzi	large B cell lymphoma	CD19	mouse FMC63	IgG4-CD28-41BB-CD3ζ	SIN lentivirus	CD4/CD8 T cells (1:1 ratio)	Wang et al.^6^; Elmacke et al.^7^; Morschhauser et al.^8^; Siddiqi et al.^9^
		chronic/small lymphocytic lymphoma						
		follicular lymphoma						
		mantle cell lymphoma						
Obecabtagene autoleucel	Aucatzyl	B cell precursor acute lymphoblastic leukemia	CD19	CAT	CD8a-CD8a-41BB-CD3ζ	SIN lentivirus	T cells	Roddie et al.^10^^,^^11^; Ghorashian et al.^12^
Idecabtagene vicleucel	Abecma	multiple myeloma	BCMA	mouse-derived scFv	CD8a-CD8a-41BB-CD3ζ	SIN lentivirus	T cells	Sharma et al.^13^
Cilatacabtagene autoleucel	Carvykti	multiple myeloma	BCMA	dual camelid single-domain binders	CD8a-CD8a-41BB-CD3ζ	SIN lentivirus	Peripheral blood mononuclear cells	Natrajan et al.^14^

Traditionally, CARs have been classified into generations based on differences in intracellular signaling features: first-generation CARs have an immunoreceptor tyrosine-based activation motif (ITAM) domain such as CD3ζ, which enables T cell activation but not sustained response; second-generation CARs pair the activation domain with one co-stimulatory domain (typically derived from CD28 or 4-1BB), which improves persistency and clinical performance; and third-generation CARs incorporate an additional co-stimulatory domain, which enhances potency in preclinical models though without clear clinical benefit.^15^^,^^16^ Despite their remarkable success in treating B cell malignancies, mounting clinical experience has revealed key limitations of classical CAR designs, including limited persistence, antigen escape, and severe toxicities.

Incremental refinements within first- to third-generation CAR architectures have partially addressed these challenges. Co-stimulatory domain selection has been proven to greatly influence CAR T cell phenotype: CD28 favors glycolytic metabolism, enhances effector phenotype, and increases expansion capacity, while 4-1BB maintains mitochondrial fitness and promotes differentiation into memory-like T cells.^17^ Antigen escape can be mitigated through careful target selection, while toxicity may be reduced through the use of fully human CAR ectodomains and vigilant clinical monitoring.^18^^,^^19^ Additional design optimizations, such as affinity maturation, modifying signaling strength through ITAM multiplicity, or further adjusting co-stimulatory signaling, have also been explored.^20^^,^^21^ Importantly, however, these strategies remain constrained within the classical CAR framework.

In contrast, next-generation CAR designs move beyond traditional CAR architectures by often integrating additional functional modules to overcome the major limitations of current therapies. Such CAR T cell products can be engineered, for example, by reshaping CAR architecture (including multiantigen, logic-gated, and universal/split receptors); introducing auxiliary genetic programs that rewire T cell fitness, trafficking, and safety; or formulating combinations of complementary CAR-engineered cell populations. Here, we refer to these as multifunctional CAR T cells.

Recent reviews have extensively characterized the great diversity of such multifunctional CAR T cells, especially regarding innovative designs tested in preclinical studies.^22^^,^^23^^,^^24^ In this systematic review, we focus on multifunctional CAR T cells that entered clinical trials, providing a comprehensive and data-driven discussion on the position of such strategies in the current clinical landscape and their trends.

## Results

### Safety and efficacy as pillars of CAR T cell design

**Figure 1:**
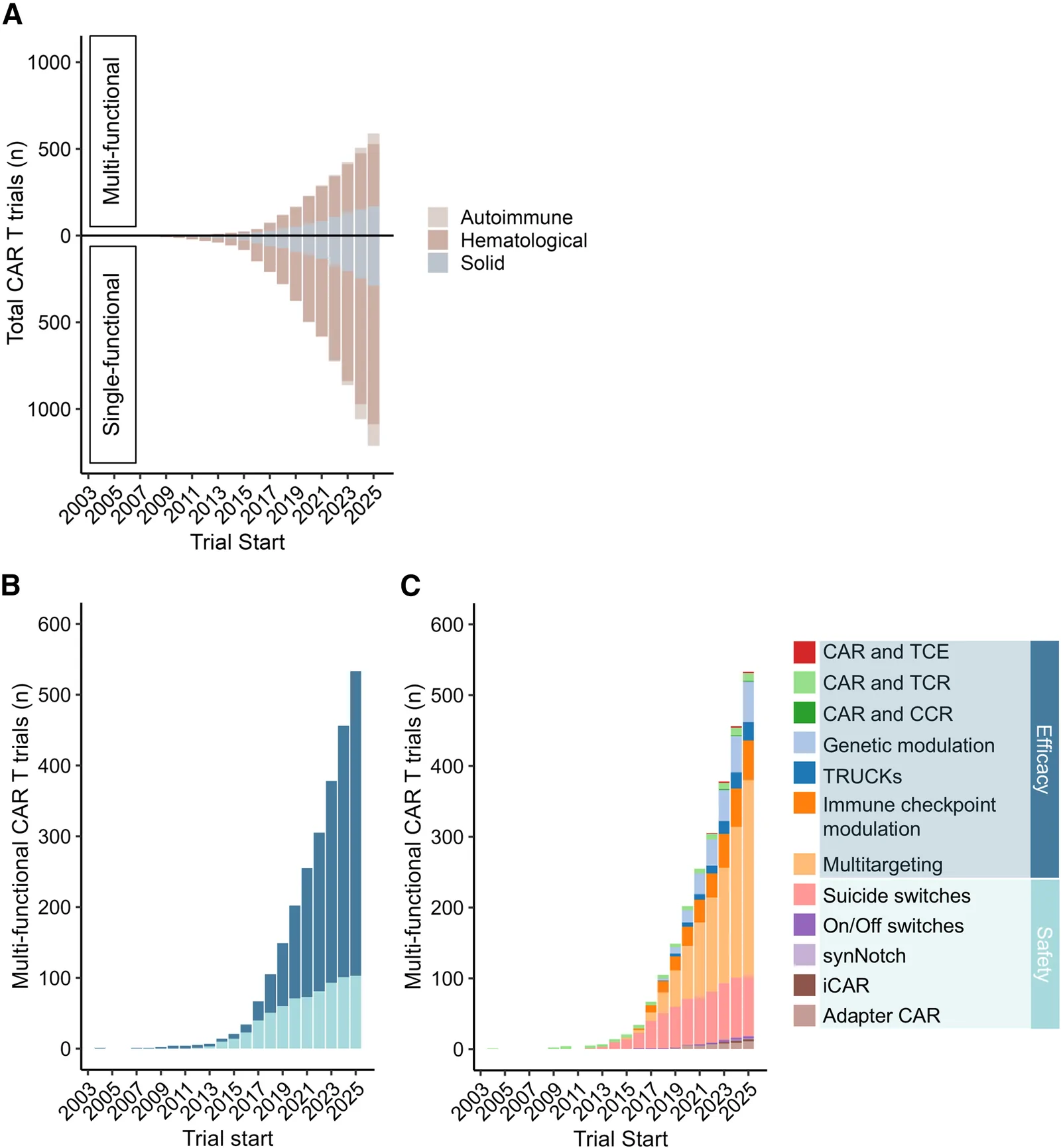
Clinical trial landscape of multifunctional CAR T cells (A) Cumulative number of clinical trials investigating single- or multifunctional CAR T cells interventions over time. Cumulative number of multifunctional CAR T cell clinical trials stratified in (B) safety- or efficacy-enhancing additional features and (C) multifunctional CAR T cell approaches.

To assess the clinical application of multifunctional CAR T cells, we curated and queried a dataset including all CAR T cell trials registered at ClinicalTrials.gov by September 2025. After screening and eligibility assessment, we included a total of 1,801 clinical CAR T cell trials (see materials and methods). Among them, we identified 533 multifunctional CAR T cell trials, labeling the remaining 1,268 as single-functional (Figure 1A). Altogether, CAR T cell therapies showed strong growth in the number of registrations per year from 2012 onward, indicating that the field is still in a period of rapid technological advancement. The subset of multifunctional CAR T cell approaches steadily increased since 2015, reaching 33% of all new CAR T cell products submitted for clinical testing in 2025.

To understand whether there are distinct preferences for clinical indication types between single- and multifunctional T cells, we further stratified the trials into hematological malignancies, solid tumors, or autoimmune diseases. Across both single- and multifunctional CAR T cell trials, hematological indications dominate (66% with 801 trials and 61% with 358 trials, respectively). Solid tumors account for a growing proportion (24% with 288 trials in single-functional and 29% with 169 trials in multifunctional CAR T cells), reflecting efforts to overcome barriers in the solid tumor microenvironment. Notably, autoimmune disease trials—though still a minority—have increased from 35 trials in 2023 to 185 trials in 2025, marking the leveraging of CAR T technologies beyond oncology to treat immune-mediated conditions.

Since the main limitations of approved CAR T cells concern safety and efficacy, many authors have previously discussed innovative CAR T cell designs across these axes.^22^^,^^25^^,^^26^ Although several enhancements in multifunctional CARs blur the lines between safety and efficacy, these dimensions remain useful analytical frameworks. Thus, we continued by subdividing multifunctional CAR T cell interventions into primarily safety or efficacy enhancing (Figures 1B and 1C). These are introduced and discussed in the following subsections.

### Designs improving safety and their clinical translation

In principle, safety-enhancing designs improve controllability over CAR activity by integrating a control module in CAR T cells. They offer either irreversible or reversible control over the CAR T cells and can be either user-controlled by drug administration or self-controlled by Boolean-logical gating.

**Figure 2:**
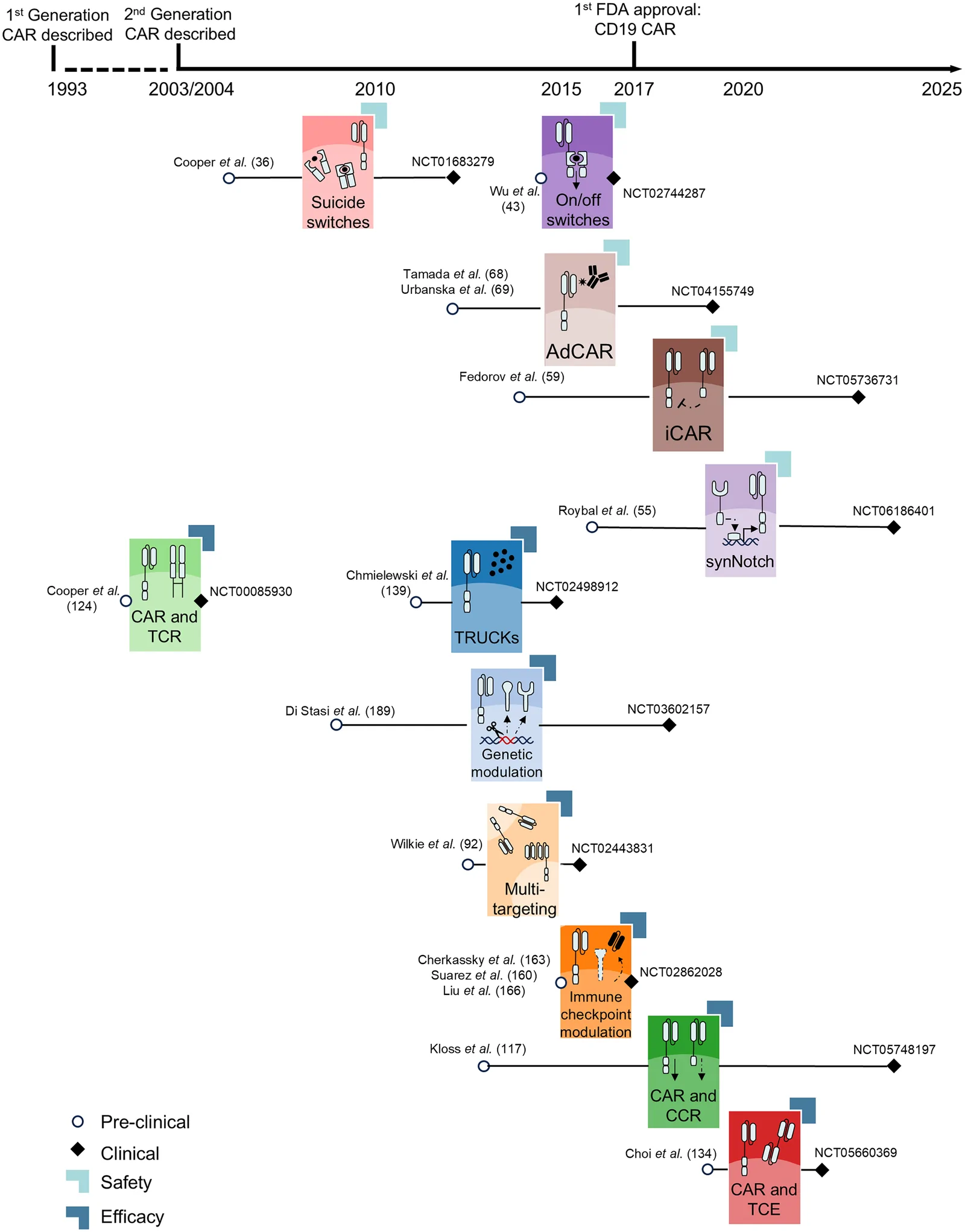
Timeline of the development of multifunctional CAR T cells First preclinical reports and first trial registrations are depicted for each technology as indicated.

A chronological view on the submissions of multifunctional CAR T trials reveals a clear priority on safety during early years (Figure 1B). One interpretation could be that, before FDA approval in 2017, the field was dominated with concerns about severe side effects such as cytokine release syndrome (CRS), immune effector cell-associated neurotoxicity syndrome (ICANS) and gene therapy’s historical setbacks.^27^^,^^28^^,^^29^ FDA approval, accumulating clinical experience, and improved toxicity management have together alleviated some of these concerns. Under this interpretation, the lack of gain in momentum of safety-enhancing designs would reflect greater confidence in risk management rather than reduced interest in safety itself. Accordingly, demands for safety enhancements remain active, as demonstrated by the clinical translation of innovative safety-enhancing designs in more recent years (Figures 1C and 2). Compared to suicide switches, the first and still most prevalent format in clinical trials, more recent designs aim to control CAR T cells’ activity rather than their elimination, underscoring the ongoing pursuit of systems that enhance safety without compromising effectiveness.

#### Suicide switches

Depletion of CAR T cells can be enabled by transducing T cells with a cassette encoding the CAR and a selection marker that leads to cell death upon exposure to specific drugs. This allows clinicians to halt the therapy in response to adverse events. Distinct mechanisms of action classify the so-called suicide switches into three main categories: inducible apoptosis switches such as inducible caspase 9 (iCasp9) trigger apoptosis when exposed to dimerizing agents (e.g., AP190)^30^^,^^31^; surface markers such as truncated EGFR,^32^ truncated HER2,^33^ or RQR8^34^ trigger antibody-dependent cell-mediated cytotoxicity (ADCC) upon administration of monoclonal antibodies such as cetuximab, trastuzumab, and rituximab, respectively; and switches such as HSV-TK disrupt DNA replication by administration of activating pro-drugs.^35^^,^^36^ Clinical trials have been addressing questions about HSV-TK switches’ increased immunogenicity risk due to virus-derived sequences^35^ and ADCC switches’ challenges with antibody tissue penetration.^37^ These critiques are not resolved despite completion of some trials using HSV-TK (NCT00730613 and NCT04097301) and ADCC switches (NCT02631044 and NCT02311621), as switches were not activated during the trials due to acceptable safety of the CAR T cells. iCasp9 switches, which are of human origin and thus less likely to be immunogenic, have not raised as many concerns.^38^ Altogether, over 100 trials are evaluating the clinical application of suicide switches, with iCasp9 and truncated EGFR being the vast majority. Of note, only two trials reported activation of the iCasp9 in two patients (NCT03016377 and NCT03373097).^38^^,^^39^^,^^40^^,^^41^ In both patients, iCasp9 was activated either after altered state of consciousness or treatment-resistant ICANs, and resulted in elimination of over 90% of CAR T cells from the circulation within one day.^39^^,^^40^ This level of depletion was sufficient to mitigate adverse events, with improvement of ICANS symptoms within four hours.^39^ Another study reported reamplification CAR T cells six weeks after iCasp9 activation, indicating incomplete elimination.^40^ While these results favor iCasp9, clinical evidence on the usage of other suicide-switch formats are required to conclusively compare them in terms of safety and efficacy.

#### On/off switches

By regulating CAR expression via administration of small molecules, on/off switches offer the advantages of reversible changes and preservation of the CAR T cell count. Conceptually, these switches come in the formats “off” and “on.” Off-switches keep CARs active under normal conditions and can be selectively “turned off” by small-molecule drugs. The CAR (or the linked signaling domain) is fused to a degradation tag, allowing compounds such as asunaprevir^42^^,^^43^ or Shield-1^44^ to trigger small-molecule-induced degradation. For example, small-molecule-assisted shutoff (SMASh)-CARs or switch-off (SWIFF)-CARs incorporate a degron and a protease motif within the CAR. Normally, the protease removes the degron and keeps CAR T cells functional, but asunaprevir prevents the protease from doing so, thereby triggering CAR degradation through the degron motif.^42^^,^^43^ Off-switch CAR T cells have not been translated to the clinic, likely because the niche of drug-mediated CAR T cell inhibition has been successfully filled by combination therapies with kinase inhibitors such as dasatinib, a readily available and well-tolerated drug that reversibly disrupts T cell signaling.^45^^,^^46^^,^^47^ Moreover, a limitation of off-switches is that CAR T cells persist long term, and continuous drug administration is impractical to control potential adverse events. On-switches circumvent this problem by keeping CARs inactive under normal conditions and “turning them on” only as long as the drug is present. Three clinical trials have evaluated on-switches, all using a format where the CAR is split into subunits that assemble into a functional heterodimeric receptor via dimerizing agents, e.g., rapalogs such as AP21967^43^ or rapamycin.^48^ Two of these trials were discontinued due to dose-limiting toxicities and the occurrence of two treatment-related deaths (NCT04650451 and NCT02744287).^49^^,^^50^ By contrast, the third study (NCT05105152) has reported early data from three patients, demonstrating activation and expansion of infused CAR T cells in the presence of the dimerizing agent rapamycin, along with a transient reduction of tumor cells.^48^ The pharmacokinetics and tolerability outcomes from this ongoing study remain eagerly awaited and might affect the translation of other on-switch formats based on small drugs that have been explored in preclinical studies, including designs based on conformational changes to an “on” state,^51^ protease cleavage of a degron attached to the CAR,^52^ and drug-induced splicing of the CAR transcript.^53^

#### SynNotch

Tumor-specific antigens are rare and often heterogeneously expressed within cancer tissue, whereas homogeneously expressed antigens are commonly also found on healthy tissue. Consequently, most candidate targets risk on-target/off-tumor toxicities.^54^ To mitigate this, some multifunctional CAR T cell strategies fragment signaling inputs to wire logic circuits that sharpen selectivity. For instance, a “prime-and-kill” circuit (“IF/THEN” gate) describes a two-step activation system that restricts CAR T cell activity to a combination of two different co-localized antigens. The first antigen is bound by a priming synthetic Notch (SynNotch) receptor, leading to the release a transcriptional activation domain that induces expression of a CAR targeting the second antigen.^55^^,^^56^

Currently, one clinical trial (NCT06186401) is evaluating SynNotch CAR T cells in glioblastoma patients. In this approach, CAR T cells are first primed via EGFRvIII (an antigen that is highly but heterogeneously expressed on glioblastoma cells) to induce expression of an EphA2/IL13Rα2 tandem CAR. While EphA2 and IL13Rα2 are broadly expressed across glioblastoma, they are also present in epithelial cells and the pituitary gland/testis, respectively.^57^ First results of this trial are awaited. In respective preclinical models, CAR expression was induced only in the presence of EGFRvIII and led to superior tumor eradication of SynNotch CAR T cells compared to CAR T cells targeting EGFRvIII or EphA2/IL13Rα2 alone. CAR surface expression declined when the priming antigen was no longer present, preventing sustained activation in antigen-negative environments.^58^ The SynNotch CAR T cells also exhibited improved persistence *in vivo*. Accordingly, investigators observed an enhanced naive/stem cell memory-like phenotype (defined as CD45RA^+^ and CD62L^+^ T cells) and reduced exhaustion (defined by co-expression of Tim-3, Lag-3, and PD-1 post manufacturing). This may be attributed to reduced tonic signaling during SynNotch CAR T cell manufacturing.^56^^,^^58^ Altogether, by integrating the recognition of multiple “imperfect” but complementary antigens, SynNotch is a promising approach to reduce on-target/off-tumor toxicity and favor memory T cell phenotype.

#### iCAR

Inhibitory CAR (iCAR) T cells constitutively express two CARs on their surface: the first recognizes a tumor-associated antigen and leads to T cell activation, whereas the second targets an antigen expressed on healthy tissue and triggers inhibitory signaling. This strategy, termed a “NOT” gate, characterizes another circuit with potential to reduce on-target/off-tumor toxicity. Several inhibitory signaling domains, including those derived from PD-1, CTLA-4,^59^ and LIR,^60^^,^^61^ have been explored for iCAR design. For this approach to be effective, the inhibitory receptors must only suppress T cell function in the presence of the respective targets. Importantly, the T cell function must recover after prior exposure to the inhibitory antigen. Since iCARs rely on antigen-specific inhibition, the chosen inhibitory ligand must be sufficiently expressed in healthy tissue to trigger an appropriate inhibitory response.

iCAR T cells under clinical evaluation leverage the loss of heterozygosity to discriminate between healthy and tumor tissue (NCT06051695 and NCT05736731). In this approach, the inhibitory receptor recognizes HLA-A∗02, which is widely expressed on healthy cells but lost in various solid tumors due to loss of heterozygosity under immune pressure.^62^ The activating CAR targets either mesothelin (MLSN)^63^ or carcinoembryonic antigen (CEA).^64^ Of note, this strategy is restricted to a particular subset of patients, as HLA loss occurs in approximately 15% of solid tumors,^62^ and about 44% of the US population are *HLA-A∗02* positive.^65^ Therefore, the success of iCAR therapy depends on carefully selecting the inhibitory receptor’s target to ensure all healthy tissues expressing the CAR antigen are adequately protected while maximizing patient eligibility.

#### Adapter CAR

Adapter CAR (AdCAR) T cell technology proposes a modular and controllable activation system. A defining feature of the so-called Adapter, Universal, or SUPRA CAR T cells is their redirection via soluble adapter molecules.^66^^,^^67^^,^^68^^,^^69^^,^^70^^,^^71^ Unlike traditional CARs, where the extracellular domain directly binds a tumor-associated antigen, AdCARs rely on soluble adapter molecules to mediate antigen specificity. This provides several advantages: precise control over AdCAR T cell activity through the administration and dosing of the adapter^66^^,^^72^^,^^73^; flexibility to switch targets by exchanging the adapter without the need for re-engineering the cell product; option to simultaneously^66^^,^^74^^,^^75^ or sequentially^76^ target multiple antigens; and potential for dual targeting of both the tumor microenvironment and tumor-associated antigens.^77^ Current AdCAR T cell trials aim to demonstrate strict adapter dependency instead of multitargeting, hence we classified the strategy as primarily safety enhancing. Several AdCAR platforms have been translated to clinical studies, including those that recognize mutated Fc regions, chemical tags, or peptide tags.

Fc-binding AdCARs recognize the Fc region of immunoglobulin G antibodies, thereby enabling the combination of CAR T cell therapy with clinically approved monoclonal antibodies. To mitigate the risk of autoimmune side effects due to AdCAR T cells engaging with endogenous antibodies,^78^^,^^79^ mutations were introduced into the Fc region of monoclonal antibodies to allow the development of CARs that specifically target the mutated Fc portion.^80^ Such AdCAR T cells are currently under clinical investigation for targeting Claudin18.2 in solid tumors (NCT05199519), where early studies have demonstrated a favorable safety profile and CAR T cell expansion.^81^

Fluorescein isothiocyanate (FITC)-specific AdCAR T cells are being evaluated in osteosarcoma patients using FITC-tagged single-chain variable fragments (scFvs) directed against the folate receptor 1 (NCT05312411). While successful engraftment of AdCAR T cells has been observed, efficacy data remain unavailable.^82^

Peptide-specific AdCAR T cells have three notable candidates under clinical evaluation. UniCAR T cells, which recognize a peptide derived from the human nuclear autoantigen La/SS-B, are redirected against prostate cancer via prostate-specific membrane antigen (PSMA) (NCT04633148) and acute myeloid leukemia (AML) via CD123-specific adapters (NCT04230265). Although UniCAR T cells redirected against prostate cancer were well tolerated, the trial was terminated due to limited biological activity.^83^ In contrast, in patients with AML, a 53% overall response rate was observed upon treatment with UniCAR T cells and CD123-specific adapters. Importantly, treatment-related toxicities were reversible upon discontinuation of the adapter molecule.^84^^,^^85^^,^^86^ Another peptide-based platform, PNE-specific AdCAR T cells, targets CD19 in B cell malignancies (NCT04488354 and NCT04450069) and has demonstrated strict adapter-dependent CAR T cell expansion.^87^ The ARC-SparX system, also in early clinical evaluation, uses a human α-fetoprotein-derived adapter and employs D domains as binding scaffolds for both the CAR and the adapter.^88^ The use of D domains has been associated with improved cell-surface expression, enhanced transduction efficiency, and reduced aggregation potential.^89^ However, synthetic scaffolds pose the risk of immunogenicity, which has been partially addressed by removing immunogenic epitopes identified through bioinformatics analysis. Nonetheless, whether these changes effectively reduce immunogenicity has not yet been tested in cellular assays.^90^ According to FDA guidelines for CAR T cell development, testing of cellular and humoral immune response against the CAR T cell product is recommended, although specific methods are not outlined.^91^

In summary, AdCAR platforms offer a highly adaptable and controlled approach to targeting complex and heterogeneous tumors. By allowing dynamic dosing and the ability to switch targeting moieties, AdCARs provide a promising strategy for improving both safety and efficacy in CAR T cell therapy.

### Designs improving efficacy and their clinical translation

Since 2016, the subset of multifunctional CAR T cells comprising efficacy enhancements quickly expanded, outpacing safety enhancements within 2 years (Figure 1B). In this context, the FDA approval of the first CAR T therapies marks a turning point in the priorities of the CAR T field that is characterized by the alleviation of primary safety concerns around CAR T therapy and the emerging goal to broaden its applicability beyond B cell malignancies. Clinical investigations made evident that solid tumors and complex hematological cancers, e.g., AML, pose significant challenges related to tumor heterogeneity, immunosuppressive environments, trafficking, and persistence of CAR T cells. Consequently, efficacy enhancements became pivotal to overcome such challenges. The pressure of unmet medical needs from many different cancer types is most likely to be the main driver of the accelerated efforts to translate potentially beneficial strategies to the clinic. Notably, such efforts have manifested not only through the rapid increase in the total number of multifunctional CAR T trials investigating efficacy enhancements but also the great diversity of strategies that are being translated (Figures 1C and 2).

#### Multitargeting CAR T cells

Targeting multiple antigens is a straightforward strategy to counteract cancer heterogeneity and antigen loss while boosting CAR T cell potency. Underscoring the growing demand for therapies addressing these points, multitargeting CAR T cells are the dominant strategy within all multifunctional CAR T trials, accounting for 52% of all such approaches in 2025 (Figure 1C). Here, multitargeting CAR T cells comprise pooled, dual, and tandem CAR T cells. The last two are typically classified as “OR” gates in the context of logic circuits, since activation is triggered by either one of the targets.

In pooled CAR T cell products, two or more CAR T cell products with different antigen recognition moieties are combined. Although conceptionally simple, this approach requires thoughtful examination of the infusion dose, timing, and order, as well as thorough assessment of potential synergy or dominance among the CAR T cell products. Alternatively, dual or trivalent CAR T cells can be engineered to express two^92^^,^^93^^,^^94^ or three^95^^,^^96^^,^^97^ different CARs on the surface of the same cell. To this end, CARs are introduced either via a bicistronic vector or co-transduction of single vectors. In contrast, tandem CARs have two scFvs linked within a single CAR protein. A tandem CAR can be designed in two ways. In the classical tandem configuration, the variable light (VL) and variable heavy (VH) chain of one scFv are directly connected to the VL and VH of the second scFv (e.g., VL1-VH1-linker-VL2-VH2). In the loop structure, the VL and VH domains from each scFv are alternately combined (e.g., VH1-VL2-linker-VH2-VL1). Although clinical results are often limited to small groups and comparisons have not shown a clear superior strategy,^98^ some preclinical studies suggest that dual CAR T cells can outperform pooled CAR T cells. For instance, dual CD19/CD123 CAR T cells in a B cell acute lymphoblastic leukemia model, as well as dual HER2/IL13Rα2 CAR T cells in a glioblastoma model, exhibited enhanced *in vivo* cytotoxicity compared to pooled CAR T cells consisting of a 1:1 mix of the respective single-targeting CAR T cells.^93^^,^^94^ In the context of HER2/IL13Rα2 and glioblastoma, another study showed even better tumor clearance by tandem CARs than by comparative dual CAR T cells, whereby improvement was hypothesized to be due to better engagement of both antigens and a denser immunological synapse.^99^ Nonetheless, it is important to acknowledge that outcomes are expected to greatly vary depending on tumor entities, antigen combinations, and binding-domain properties, even more so for clinical studies.

**Figure 3:**
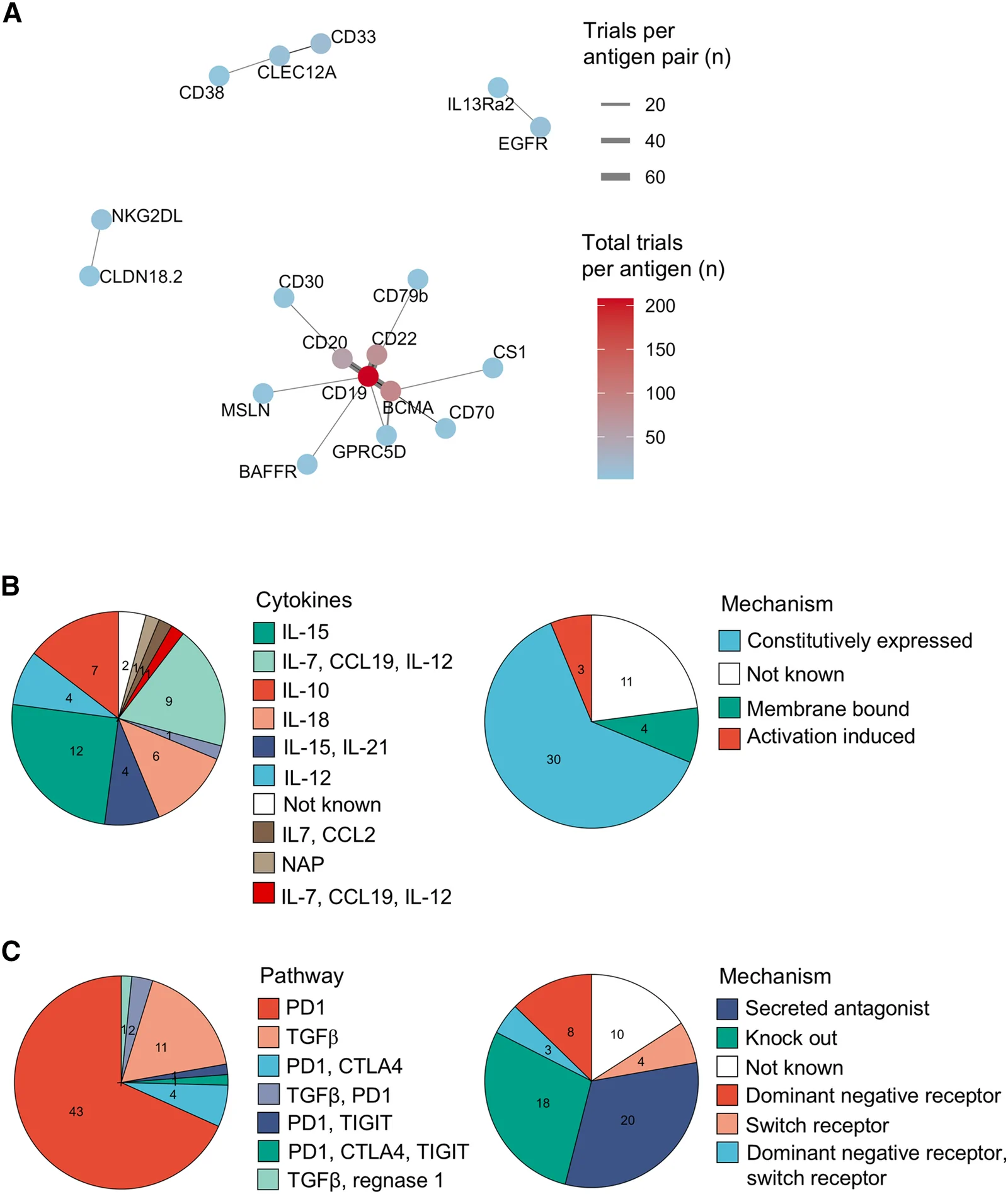
Target combinations, immune checkpoint modulation, and cytokine engineering in multifunctional CAR T cell clinical trials (A) Network of the top 20 specificities from multitargeting CAR T cells. (B) Breakdown of clinical trials based on the cytokine used for TRUCKs and the specific mechanism used for cytokine secretion. (C) Breakdown of clinical trials based on the targeted immune checkpoint pathway and the specific mechanism used for modulation.

To identify prevailing antigen combinations among multitargeting CAR T cells, we performed a network analysis that revealed four major target clusters (Figure 3A, and see materials and methods). The first cluster—CD19, CD20, CD22, and B cell maturation antigen (BCMA)—primarily targets B cell malignancies; the second—CD33, CD38, and CLEC12A—represents key AML targets; and the third and fourth—NKG2DL and CLDN18.2, EGFR and IL13Rα2—addresses solid tumors. Approximately two-thirds of clinical trials investigating multitargeting CAR T cells combine CD19 with either CD20, CD22, or BCMA. A systematic meta-analysis by Nguyen and colleagues concluded that dual-targeting CD19/CD22 CAR T cells demonstrated superior outcomes in patients with relapsed/refractory B cell malignancies compared to single-targeting CAR T cells.^100^ Similarly, a phase 1/2 trial in relapsed/refractory non-Hodgkin lymphoma observed prolonged median progression-free survival with CD19/CD20 dual CAR T cells compared to a previous trial utilizing CD19 CAR T cells alone.^101^ Dual-targeting BCMA/CD19 CAR T cells demonstrated an impressive complete response rate exceeding 82% in treated patients.^102^ Within the AML-related cluster, one clinical trial employing dual-targeting CD33/CLEC12A CAR T cells reported first results: seven out of nine treated patients achieved measurable residual disease negativity and proceeded to hematopoietic stem cell transplantation.^103^ Interestingly, a non-responder exhibited CD33 positivity but lacked CLEC12A expression.

Despite promising outcomes, defining an optimal target combination remains a critical challenge. Ideal targets should provide broad tumor coverage to minimize the escape risk of clonal variants, exhibit sufficient specificity to reduce on-target/off-tumor toxicities, and demonstrate stability to ensure CAR engagement.^104^ Advancements in single-cell transcriptomics and proteomics have enabled more precise target selection for CAR T cells,^105^^,^^106^^,^^107^ and are now being leveraged to identify suitable pairs for multitargeting strategies.^108^^,^^109^ Hu and colleagues developed a cancer surfaceome atlas, uncovering 179 unique antigen pairs with potential application for multitargeting CAR T cells.^107^ Antigen selection should also account for the physical properties of target antigens. For instance, CAR T cells with short hinge regions perform better against membrane-distal epitopes, while longer hinge regions are more effective against membrane-proximal epitopes.^110^^,^^111^^,^^112^^,^^113^ Hence, linker length in tandem CAR constructs are deemed to impact therapeutic efficacy. Moreover, antigen density may establish distinct preferences for co-stimulatory domains.^114^^,^^115^^,^^116^ Taken together, insights from single-target CAR constructs should be considered in the design of multitargeting strategies.

#### CAR and CCRs

Chimeric co-stimulatory receptors (CCRs) consist of an extracellular binding domain, a spacer, a transmembrane domain, and a co-stimulatory domain. Unlike CARs, CCRs lack an ITAM-containing signaling domain such as CD3ζ; meaning they do not trigger cytotoxicity upon antigen binding but instead provide additional co-stimulation when paired with CARs (“IF/BETTER” gates).^117^ Therefore, CCRs can increase CAR T cell sensitivity against tumor cells with low CAR antigen expression and mitigate antigen escape. To ensure balance between enhanced tumor recognition and minimal on-target/off-tumor toxicities, CAR and CCR affinities as well as target antigen selection require careful consideration; for example, healthy cells should express the CAR target at low levels and must not express the CCR target.

Currently, one clinical study is evaluating the combination of ADGRE2 CAR and CLEC12A CCR in AML patients (NCT05748197). In respective preclinical models, CCR addition enabled the killing of ADGRE2^low^ CLEC12A^pos^ AML cells while sparing ADGRE2^low^ CLEC12A^neg^ healthy hematopoietic stem cells.^118^

Meanwhile, several preclinical studies have provided further valuable insights into CAR and CCR combinations: first, using the same hinge and transmembrane domains for both CARs and CCRs can ensure optimal tumor-dependent co-stimulation; second, direct comparisons show that co-stimulation in *trans* (co-expression of separate chimeric receptors) is more effective than co-stimulation in *cis* (where both signals are integrated within a single third-generation CAR)^119^^,^^120^; third, CCR integration reduces the overall T cell activation threshold^119^^,^^120^^,^^121^^,^^122^; and fourth, different types and quantities of the CCR co-stimulatory domains impact the synergy between CAR and CCR signaling.^119^^,^^120^^,^^121^^,^^122^

#### CAR and TCRs

Leveraging the activation signals of native T cell receptors (TCRs) to improve efficacy of CAR T cell therapy was proposed over 20 years ago^123^ and is the very first multifunctional CAR T cell strategy to be recorded in clinical trials (NCT00085930 and NCT01109095). For this, virus-specific T cells were used as starting material for CAR T cell manufacturing.^124^ Emblematic examples include Epstein-Barr virus (EBV), cytomegalovirus (CMV), and adenovirus.^125^^,^^126^ Since these are common in most people, the strategy holds promising medical relevance, especially in the light of recently approved allogeneic EBV-positive T cell therapy.^127^ At its rationale, TCR reactivation by the virus within the patient could provide additional stimulus to the CAR T cells, potentially enhancing persistence and anti-tumor activities. To date, 10 such trials have been registered. Lapteva et al. demonstrated that multivirus-specific CD19 CAR T cells expanded rapidly in response to viral load, whereas their proliferation was reduced in patients without pre-existing EBV, CMV, or adenovirus infection (NCT00840853).^125^ Conversely, long-term follow-up of GD2 CAR T cells in neuroblastoma patients revealed that that when both conventional GD2 CAR T cells and EBV-specific GD2 CAR T cells were infused, the conventional CAR T cells were more frequently detected at later time points (NCT00085930). Whether this difference is attributed to inadequate TCR engagement or difference in T cell homing remains unclear.^128^ To further explore the potential of virus-specific CAR T cells, 2 ongoing clinical studies are evaluating the use of therapeutic vaccines to stimulate the virus-specific TCRs in such CAR T cells *in vivo* (NCT05801913 and NCT05432635).

Although not yet in clinical trials, many preclinical studies have been combining CARs with transgenic TCRs to enable dual targeting of tumor-associated antigens. The concept has been tested by pooling separate CAR T cells and transgenic TCR T cell products,^129^ as well as incorporating both receptors into the same T cell.^130^^,^^131^ For the latter, conclusions were controversial as to whether the CAR and transgenic TCR synergize in signaling. More recently, Kondo and colleagues used empirical data to mathematically model the CAR-TCR crosstalk, concluding that weak TCR-antigen interactions antagonize CAR signaling, whereas strong TCR-antigen interactions boost it.^132^ While the model may partially solve controversies, it is still unclear how CAR-TCR combinations can be best used. The authors demonstrated that certain TCRs with strong affinity for neoantigens often weakly recognize self-antigens. Thus, they propose leveraging the combination of such TCRs with CARs to both dampen CAR on-target/off-tumor toxicity and simultaneously enhance the killing of cancer cells expressing high-affinity neoantigens. On this note, target antigen selection and HLA backgrounds should be carefully considered to shape strategies that maximize a patient’s eligibility.

While preclinical and early clinical studies suggest promising benefits for both endogenous and transgenic TCR-based CAR T cell strategies, further investigation is needed to determine their full therapeutic potential. The endogenous TCR approach may be particularly beneficial in settings where viral antigens can provide additional stimulation, whereas transgenic TCRs could offer a dual-targeting strategy in the future.

#### CAR and TCEs

Bispecific T cell engagers (TCEs) are recombinant proteins comprising two tandem scFvs: one targeting the tumor antigen and the other binding CD3 on the T cell surface. IL13Rα2 CAR T cells secreting anti-EGFR TCEs demonstrated faster tumor clearance *in vivo* compared to IL13Rα2 CAR T cells alone. However, their efficacy was surpassed by dual CAR T cells and T cells secreting dual TCEs bearing the same specificities.^133^ When choosing the targets wisely, the combination of CAR and TCEs allows targeting of a tumor-associated antigen via the CAR and a second antigen via the TCEs, which otherwise would be considered undruggable due to on-target/off-tumor toxicity. Choi et al. demonstrated in preclinical models that TCEs secreted by the CAR T cell act locally, thereby minimizing systemic exposure and improving the safety profile compared to TCE therapy alone. Overall, EGFRvIII CAR T cells secreting EGFR-TCEs demonstrated tumor clearance, while EGFRvIII CAR T cells failed to control tumor growth *in vivo*.^134^ An early phase 1 trial reported no severe adverse events; however, anti-tumor responses were transient, potentially due to the limited persistence of CAR T cells.^135^

#### TRUCKs

Cytokine signaling, the classic signal 3 of T cell activation, is critical for proliferation and differentiation to effector cells. CAR T cells struggle to deliver a diversified signal 3^136^ and, while augmenting it can enhance persistence and tumor infiltration, systemic cytokine delivery risks high toxicities.^137^^,^^138^ T cells redirected for universal cytokine-mediated killing (TRUCKs) co-engineer CARs with cytokine genes to concentrate signal 3 within tumors.^139^ As we focus on the most prevalent cytokines in TRUCK trials, we refer the reader to recent reviews that extensively cover the general diversity of cytokine armors.^140^^,^^141^

In clinical settings, interleukin-15 (IL-15) represents the most frequent cytokine payload, followed by IL-10 and the combination of IL-7/CCL19 (Figure 3B). Regarding expression patterns, most clinical TRUCKs rely on constitutive cytokine secretion; few membrane-bound formats appear in IL-15 TRUCK trials aiming at reducing systemic exposure; and two trials (NCT03542799 and NCT06002659) have employed activation-induced secretion systems to restrict cytokine release to CAR engagement (Figure 3B).

The use of IL-15, a key cytokine in T cell homeostasis and survival, improved CAR T cell potency and persistence in many preclinical models of liquid and solid tumors^142^^,^^143^^,^^144^ and is currently present in 16 trials investigating IL-15 and IL-15/IL-21 TRUCKs. Notably, engineering T cells with IL-15 has also induced preleukemic disorder in mice,^145^ reflecting on the incorporation of suicide switches by most of the IL-15 TRUCK trials. Recently, first clinical results became available: in a side-by-side comparison between IL15-GPC3 TRUCKs and GPC3 CAR T cells in various solid tumors (NCT05103631 and NCT04377932), the comparative TRUCK groups showed enhanced anti-tumor responses and peak expansion as well as a manageable safety profile despite the higher incidence of CRS.^41^ Other IL-15 TRUCK trials have not yet published their results. These include variations such as membrane-bound IL-15 and combination of IL-15/IL-21, both of which are supported by preclinical findings demonstrating enhanced T cell memory phenotype^146^^,^^147^ and improved tumor control,^148^ respectively.

Recent preclinical studies showed that IL-10 secretion can restore exhausted T cells,^149^ leading to trials currently evaluating the safety and efficacy of IL-10 TRUCKs in patients with hematological malignancies. First results on IL10-CD19 TRUCKs demonstrated a manageable safety profile and a higher complete response rate compared to that of commercially available CD19 CAR T cells (NCT06277011).^150^

IL-7/CCL-19, the most prevalent duo in clinical trials, supports dendritic- and T cell recruitment to lymph nodes. Since preclinical studies demonstrated that IL-7/CCL19 secretion increases tumor infiltration,^151^^,^^152^ multiple TRUCK trials have implemented the combination, with many trials reporting promising results on safety and efficacy in different liquid and solid tumor indications.^153^^,^^154^^,^^155^

Pleiotropic cytokines such as IL-18 and IL-12 promote acute inflammation in the tumor microenvironment and increase anti-tumor activity. First clinical results on IL-18 TRUCKs have diverged: IL18-CD19 TRUCKs yielded durable remissions and manageable CRS in patients with relapsed non-Hodgkin lymphomas (NCT04684563),^156^ while IL18-CD371 TRUCKs found dose-limiting toxicities in patients with relapsed AML (NCT00466531).^157^ Although therapy outcome is highly dependent on tumor entities and primary CAR targets, these findings warrant further investigation on the safety of IL-18 TRUCKs. Similar toxicity outcomes of IL-12 TRUCKs^158^ prompted inducible IL-12 systems regulated by the NFAT promoter (NCT03542799),^159^ which are now under way to be evaluated in clinical trials.

Taken together, early clinical results confirm that different cytokines enhance CAR T function in distinct ways. They also exposed toxicity challenges unforeseen by preclinical models. Given the complexity of signal 3 and the varying needs of different tumor entities, a single dominant cytokine for TRUCKs is unlikely for the future. Instead, recent developments indicate that a consensus focus rather lies in improving control over cytokine secretion by TRUCKs.

#### CAR and immune checkpoint modulation

CAR T cell therapy often faces limitations due to T cell exhaustion, a dysfunctional state caused by prolonged antigen stimulation and immunosuppressive factors within the tumor microenvironment. To counteract this, various multifunctional CAR T cell strategies have been developed to modulate immune checkpoints, thereby reinvigorating CAR T cell function and persistence. These include secretion of inhibitory receptor antagonists,^160^^,^^161^^,^^162^ expression of truncated dominant-negative inhibitory receptors,^163^ inhibitory receptor knockdown/knockout,^164^^,^^165^ and rewiring of inhibitory receptor to transmit activating signals.^166^^,^^167^ Among these strategies, modulation of the PD-1/PD-L1 axis has been the most widely investigated in clinical trials, followed by targeting of transforming growth factor β (TGF-β) (Figure 3C). Noteworthy, only a few trials have explored the modulation of multiple pathways. While there is a clear trend in the pathways being targeted, the mechanisms of modulation vary, with secretion of antagonists and inhibitory receptor knockout being the most prominent approaches (Figure 3C).

Preclinical studies suggest that localized secretion of PD-1/PD-L1 antagonists may be more effective than systemic checkpoint blockade.^161^^,^^162^ Accordingly, early-phase clinical trials evaluating MLSN CAR T cells engineered to secrete PD-1 nanobodies have shown promising results, with detectable anti-PD-1 nanobodies in peripheral blood (NCT04503980, NCT05089266, and NCT03615313).^168^ Another strategy to counteract immune suppression involves dominant-negative receptors, in which truncated, non-signaling variants of inhibitory receptors such as PD-1 or TGF-β^163^^,^^169^^,^^170^ compete for ligand binding without transmitting inhibitory signals. This approach has demonstrated promising preclinical efficacy and is now under clinical investigation. Early trials indicate a manageable safety profile and signs of biological activity; however, long-term efficacy remains to be determined.^171^^,^^172^

Alternatively, genetic knockout of inhibitory receptors such as PD-1 renders CAR T cells resistant to PD-L1-mediated suppression in the tumor microenvironment, thus potentially enhancing expansion and anti-tumor activity. While clinical trials have demonstrated the safety of PD-1 knockout CAR T cells, efficacy has varied depending on tumor entities.^173^^,^^174^^,^^175^ Wang and colleagues suggested that limited efficacy could be attributed to low persistency of MLSN CAR T cells with PD-1 knockout: although most tumor biopsies taken 2 weeks post treatment detected CAR T cells, they became undetectable after 3–4 weeks, indicating successful tumor trafficking despite poor long-term persistence.^175^

Synthetic switch receptors translate the binding of an immunosuppressive molecule into an activation signal. To this end, some switch receptors exchange the endodomains of receptors binding immunosuppressive cytokines (e.g., IL-4) with those binding pro-inflammatory cytokines,^176^^,^^177^ while others fuse the intracellular activating domain of CD28 or 4-1BB with ectodomains of inhibitory receptors such as TIM-3,^178^ PD-1,^166^^,^^167^ or TGF-β.^179^ CAR T cells equipped with PD-1/CD28 switch receptors are being investigated in trials targeting either PSMA (NCT06046040 and NCT05489991) or CD19 (NCT03932955 and NCT04850560). Notably, a case report described a patient with diffuse large B cell lymphoma who had failed initial CD19 CAR T cell therapy but achieved sustained complete remission after receiving a CD19 CAR incorporating a PD-1/CD28 switch receptor (NCT04850560).^180^

So far, it is not clear which pathway modulation or method has the greatest clinical effect. Systematic evaluations and/or meta-analyses considering checkpoint pathway, mechanism of action, tumor types and CAR T cell dosage could give valuable insight in the field.

#### CAR and genetic modulation

This section jointly discusses further genetic modifications in CAR T cells that fall outside previous categories. Generally, these “other” strategies revolve around three goals: leveraging cytokine or chemokine receptors (16 trials), resisting lymphodepletion or fratricide (19 trials), or enhancing overall cell fitness (12 trials). Owing to our safety/efficacy scope, we exclude allogeneic approaches and refer the reader to other reviews covering the topic.^23^^,^^181^^,^^182^

First, compared to TRUCKs, combinations with constitutively active cytokine receptors bypass cytokine supply to avoid bystander immune activation, thereby restraining signal 3 to CAR T cells. The most studied example is the constitutively active IL-7 receptor (C7R).^183^^,^^184^ In a trial comparing GD2 CAR T and C7R-GD2 CAR T cells in patients with brain tumors, the comparative C7R group had prolonged improvement of neurological deficits and manageable safety despite higher incidence of CRS (NCT04099797).^185^ Alternatively, synthetic cytokine receptors that exclusively bind a cognate synthetic cytokine (orthogonal receptors)^186^^,^^187^ enable precise, on-demand signaling. First results on orthogonal IL-2 CD19 CAR T cells in relapsed non-Hodgkin lymphoma patients reported favorable safety and efficacy (NCT05665062).^188^ In addition, overexpressing chemokine receptors such as CXCR2, CCR4, CXCR4, and CXCR5 enhances CAR T cell migration into cancers, a strategy also commonly named “self-driving CAR.”^189^^,^^190^^,^^191^^,^^192^ Early results from a CCR4-CD30-CAR T trial in T cell lymphoma patients showed promising safety and efficacy (NCT03602157).^193^ Further trial results are awaited.

Second, some modifications allow CAR T cells to treat T cell malignancies without triggering self-killing (fratricide). One such strategy targets T-cell-specific antigens such as CD5 or CD7 while genetically deleting them in CAR T cells. So far, several trials have demonstrated high response rates and acceptable safety.^194^^,^^195^^,^^196^ Another promising approach within this category consists of co-expressing CARs with CD7 expression blockers,^197^^,^^198^^,^^199^ with encouraging first trial results.^200^^,^^201^

Third, investigators have identified multiple gene modifications that enhance CAR T cell fitness. For instance, overexpression of transcription factors such as c-Jun and RUNX3 yielded favorable CAR T cell phenotypes in preclinical models and are now under clinical investigation.^202^^,^^203^ First results on RUNX3 GPC3 CAR T cells in hepatocellular carcinoma patients showed promising efficacy and manageable safety profiles (NCT03980288).^203^ Similarly, overexpression of co-stimulatory receptors and agonists such as CD40L, OX40, and CD27 are also being investigated in trials,^204^^,^^205^^,^^206^ with promising first trial results by CD27 BCMA-CAR T in patients with relapsed multiple myeloma (NCT04706936).^207^ Several candidates for genetic deletion, such as HPK1, Regnase-1, and CBLB, have also enhanced CAR T fitness in preclinical models and are now under clinical investigation.^208^^,^^209^^,^^210^ Recently, a multiedited CD19 CAR T cell featuring Regnase-1 deletion showed encouraging efficacy and safety in an early trial (NCT05643742).^211^ Further results have not yet been published.

Noteworthy, gain- and loss-of-function screens have respectively identified many more candidates for overexpression^212^^,^^213^^,^^214^^,^^215^^,^^216^ and disruption^217^^,^^218^^,^^219^^,^^220^^,^^221^^,^^222^^,^^223^^,^^224^^,^^225^ that enhance CAR T cell fitness. Compared to any other multifunctional CAR T cell strategies, the candidates for genetic modulation identified in preclinical studies largely outweigh those translated to clinical studies^23^^,^^226^: a recent overview on next-generation CARs identified 28 gene modifications aimed at enhancing fitness, out of which only four have been translated to the clinic thus far.^23^ At this point, we can only speculate on the underlying reasons for this discrepancy: insufficient evidence against long-term malignant CAR T cell transformation, unmatched throughput of preclinical screening studies, and/or too recent findings.

### When different enhancing features overlap

**Figure 4:**
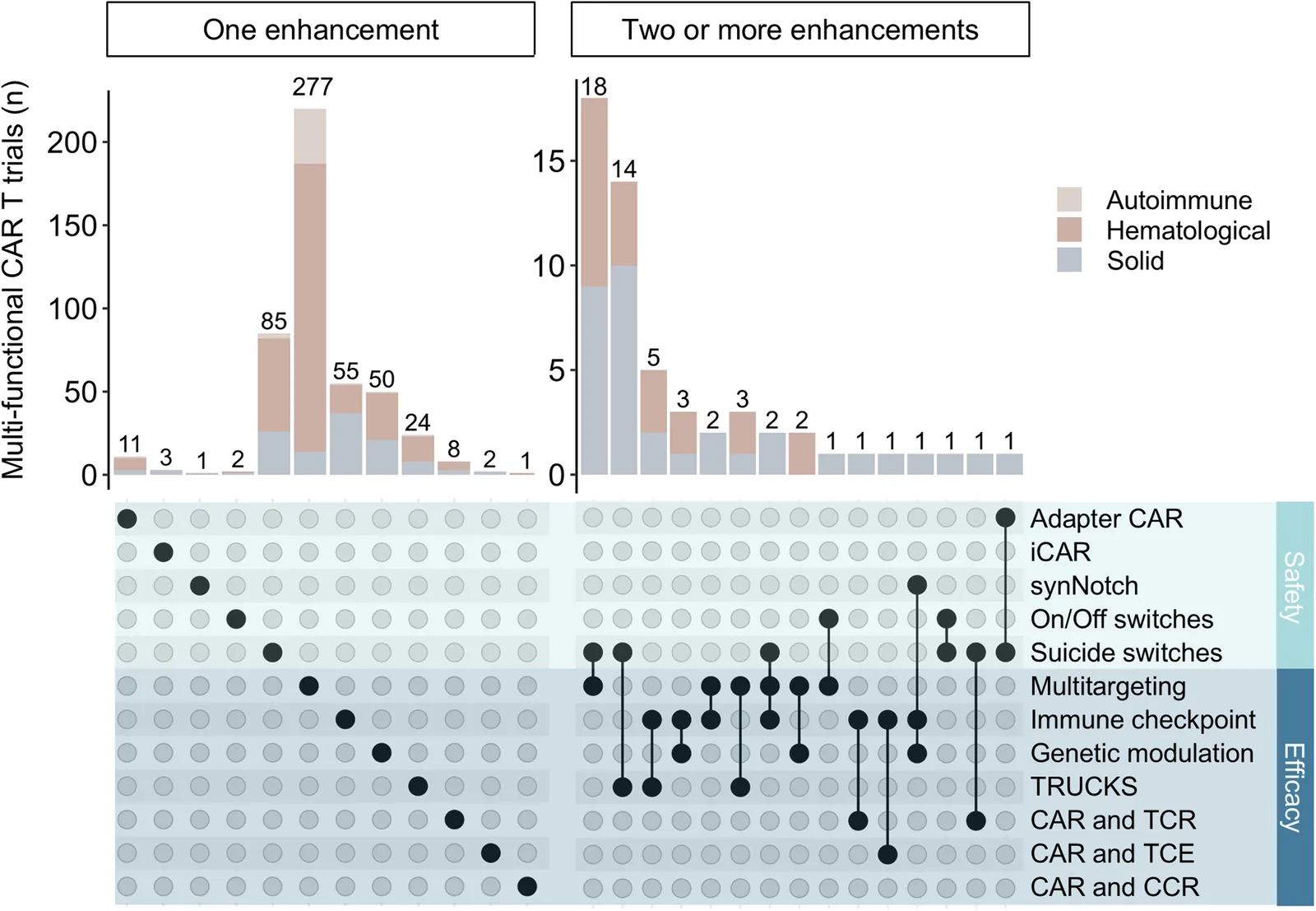
Clinical trial landscape of multifunctional CAR T cells employing one or multiple enhancing features UpSet plot showing number of multifunctional CAR T cell trials that employ one (left) or more (right) enhancing features. Bars represent the intersection of indicated features, and colors represent different types of clinical indications.

Thus far, we have discussed individual multifunctional CAR T cell strategies that equip CAR T cells with one additional feature that primarily enhances either safety or efficacy. While some approaches such as multitargeting, suicide switches, and TRUCKs have shown promising results in clinical trials, a single additional feature added to a CAR T cell may not be sufficient to address multiple barriers limiting CAR T cell efficacy and safety. Antigen escape, T cell exhaustion, immunosuppressive signals, and systemic toxicity each require distinct countermeasures. To assess whether different efficacy- and safety-enhancing features are combined in current clinical strategies, we generated an UpSet plot to visualize the frequency and co-occurrence of multiple CAR T cell modifications (Figure 4).

Most trials employ a single enhancement strategy, mainly in hematological malignancies, with efficacy-focused approaches such as multitargeting dominating. In contrast, trials combining multiple enhancements are relatively rare and more frequently associated with solid tumor indications, reflecting higher biological and translational complexity. A total of 56 clinical trials are investigating CAR T cells equipped with two or more enhancing features. Within this group, a consistent pattern emerges in which efficacy-oriented strategies, such as multitargeting or TRUCK designs, are frequently paired with safety-enhancing features, most commonly suicide switches. As discussed, most clinical studies reporting initial results did not trigger the safety switch,^158^^,^^227^^,^^228^^,^^229^^,^^230^ suggesting that their inclusion serves more as a precautionary measure than a necessity. Nonetheless, this pairing reflects a deliberate effort to balance increased anti-tumor potency with the need for improved control and safety. Notably, in this multienhanced CAR T cell subset, there is a marked increase in the representation of solid malignancies, underscoring the greater complexity of solid tumor indications compared with hematological diseases (i.e., tumor microenvironment, immune evasion, and CAR T cell trafficking to the tumor site).

Clinical trials combining a total of three efficacy- and safety-enhancing features are still uncommon, although some studies are exploring this direction (NCT05141253 and NCT05694364). This low incidence may be attributed to increased manufacturing complexity, regulatory challenges, and/or the need for carefully balancing therapeutic benefit and potential risk. In the future, it will be particularly interesting to see whether CAR T cells equipped with three or more enhancements (e.g., two efficacy-enhancing features alongside one safety mechanism) can outperform those incorporating a single or two modifications. Nonetheless, recent reports of rare malignant transformation following CAR T cell therapy have heightened concerns about genotoxicity,^231^^,^^232^ suggesting that strategies relying on non-genetic modifications to improve CAR T cell fitness may ultimately prove more competitive than extensive multiplex genetic editing.

Overall, the analysis highlights a preference for clinically tractable single-enhancement approaches and illustrates the trade-off between increasing functional complexity and clinical feasibility.

### Navigating regulatory frameworks

By implementing additional features, multifunctional CAR T cells increase study complexity, thereby creating considerable challenges for clinical translation. For instance, switch-based platforms involve additional drug administration that requires separate safety and dose assessment; pooled CAR T cells must consider timing, order, and dose when infusing different products; and CAR-TCR combinations must carefully consider the patients’ HLA backgrounds. An inherent challenge underlying all multifunctional CAR T cells is determining the best product (version) for clinical studies when so many different variables are at play, e.g., antigen combinations, co-stimulatory domains, and mechanism of expression. Since preclinical models are often not established and insufficient to reflect the complex interplay of cancer cells, microenvironment, and immune cells, it is plausible to occasionally entrust early clinical studies to elect from among the best candidates for later trial phases. However, planning comparative study arms for this purpose is further challenged by regulatory guidance being traditionally meant for powering study arms to reveal statically significant differences—not for insights into candidate selection.

To make regulatory frameworks more efficient for the study of multiple versions of cell and gene therapies, the FDA guidance adopted the “parent-child” approach in 2022.^233^ The concept proposes that the regulatory submission of a first product version (parent drug) can support subsequent versions (child drug) through cross-referencing of common sections, allowing sponsors to streamline regulatory submissions at early phases—and with defined guidelines on how to do so. While the strategy has been successfully applied in the United States, it is not directly transferable to Europe due to structural differences in the regulatory systems. The European Medicines Agency (EMA) has provided some tools within the European regulatory framework to facilitate the assessment of different product versions as well.^234^^,^^235^^,^^236^ However, EMA has so far no consolidated recommendation or guidelines on the matter, which would be important to facilitate clinical translation of advanced cell therapies such as multifunctional CAR T cells in Europe. Meanwhile, T2EVOLVE, a European consortium aimed at accelerating the development and regulatory approval of engineered T cell therapies for cancer, has provided some recommendations that could help investigators navigate the complex European regulatory framework.^237^ Altogether, recent efforts from regulatory bodies and consortia reflect the profound clinical potential of advanced cell therapies and highlight the pressure for more agile regulatory pathways.

## Discussion

Next-generation CAR T cells denote products bearing hallmarks that cannot be described as small incremental changes to first-, second-, or third-generation designs but, rather, paradigm shifts. Typically, they incorporate additional molecular modules that offer new functional features, herein referred to as multifunctional CAR T cells. Preclinical studies have shown a fast-growing pool of such innovative designs, whereby reviews frequently share updated lists that include recently reported technologies. By contrast, the clinical translation status of each technology often remains unclear.

In this systematic review, we specifically address this gap by surveying registration entries of CAR T cell studies at the Clinicaltrials.gov database. Albeit limited to the registries of one database, our approach allowed for clear visualization of the frequencies of multifunctional approaches among all CAR T clinical trials, which in turn we extrapolated as a general footprint for a semi-quantitative assessment on the clinical advances of multifunctional CAR T cells. The approach allowed for valuable insights into the field.

First, efficacy-driven multifunctional CAR T cells have grown exponentially, while safety-focused strategies have followed a logistic growth pattern (Figure 1B). For both, inflection points approximately co-localize with the 2017 first FDA CAR approval, reinforcing the meaning of this breakthrough event as a general acceptance of the therapeutic potential of CAR T cells and the interest in broadening their application to diverse indications. Notably, multifunctional CAR T cell development builds on safety knowledge established with first- to third-generation CAR T cells (single functional), so we cannot exclude their part in explaining the reduced momentum of safety-focused designs. Although functional enhancements are often motivated by challenges posed by solid tumors, only a modest enrichment of solid-tumor indications was observed in multifunctional trials compared to single functional CAR T cells (Figure 1A). By contrast, CAR T cells incorporating multiple enhancements showed a marked shift toward solid malignancies, more clearly reflecting the gained complexity of solid over heme indications (Figure 4). Another trend observed was that multifunctional CAR T trials in autoimmune diseases have shown a marked increase, particularly for multitargeting approaches.

**Figure 5:**
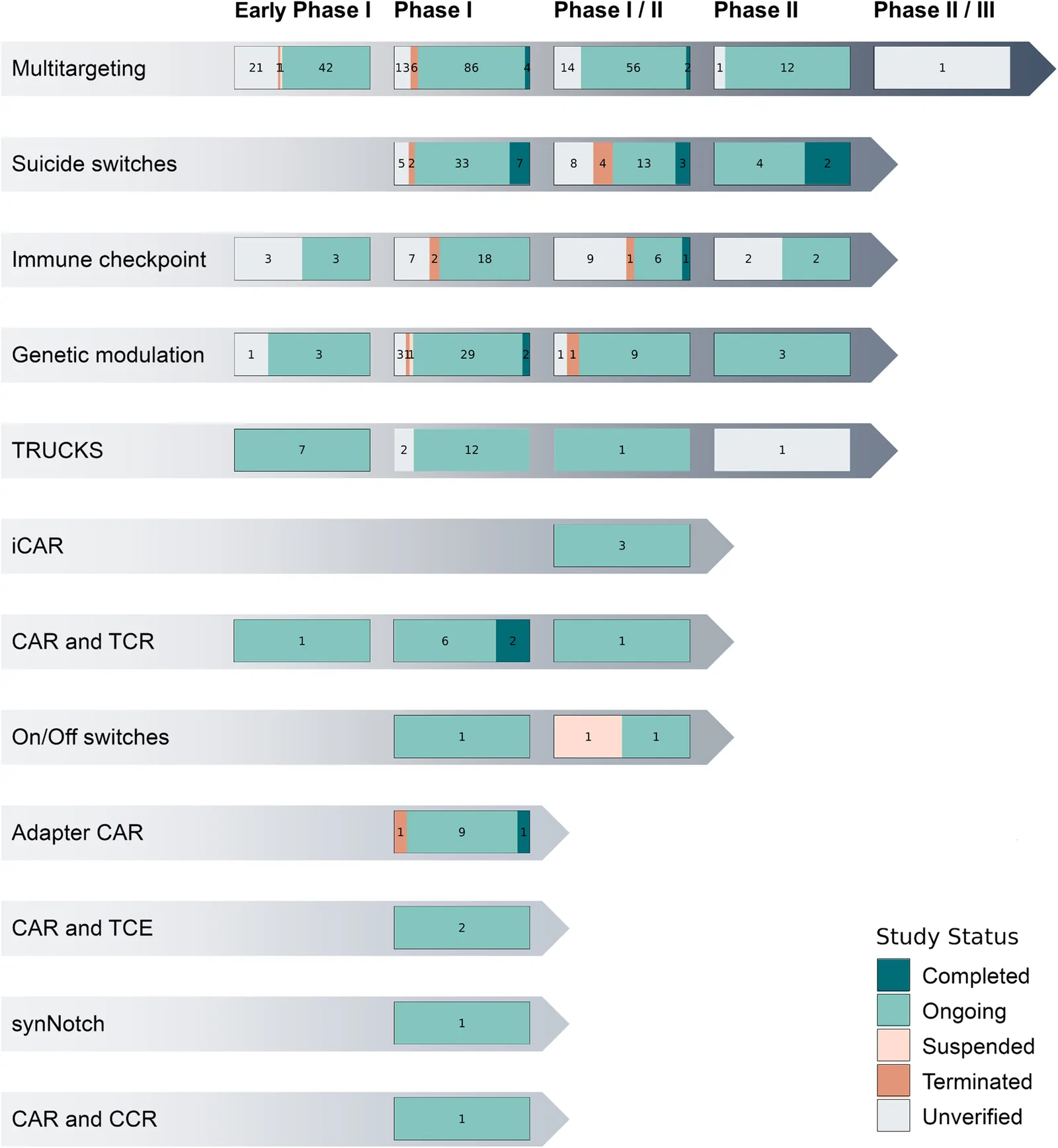
Clinical readiness status of different multifunctional CAR T cells Distribution of clinical trials evaluating multifunctional CAR T cells categorized by phase and status of the studies. Unverified studies comprise studies that passed completion date and whose status has not been last verified within the past 2 years. Early phase 1 describes exploratory trials conducted before traditional phase 1 studies.

Second, multitargeting CAR T cells constitute the largest share of multifunctional designs and are over-represented in later-phase trials, indicating advanced clinical readiness with several studies in phase 2 (Figure 5). Importantly, in our survey, multitargeting comprised pooled, dual, and tandem CAR T cells. Despite differences, the three share unresolved challenges in rational antigen selection. Moreover, tandem CARs additionally require structural optimization workflows to efficiently screen for functional construct configurations. Recent developments show that both challenges have a lot to gain from computational approaches, particularly prediction tools. On the side of antigen combination, recent advances integrating omics data and machine-learning-based algorithms have allowed for both ranking of cancer targets and prioritizing antigen pairs for CAR development.^107^^,^^108^^,^^109^ On the side of tandem designs, studies leveraged computational mutational scanning and structure prediction tools to better identify and rescue dysfunctional regions of CAR constructs.^238^^,^^239^^,^^240^ Altogether, while multiple studies testify the value of computational tools in accelerating CAR development, they have also shown that such tools alone are often insufficient to achieve biologically sensible results or reduce screening pools into manageable sizes—for this, human critical input is required. Importantly, human input is, however, currently constrained by limited understanding of key determinants of antigen synergy—such as co-localization, abundance, and topology—as well as that of CAR expression and regulation. Thus, advancing the field will most likely require integrating future CAR design libraries and mechanistic studies. Similar to ATLAS studies, meta-analyses could provide valuable insights into the matter. Yet, as we have also experienced ourselves during the preparation of this review, such analyses may be currently hindered by inconsistent nomenclature and limited data standardization of current databases, underscoring the need for more harmonized reporting frameworks.

Finally, other multifunctional CAR T cell designs remain at earlier stages of clinical development (Figure 5). Suicide switches, immune checkpoint modulation, genetic modulation, and TRUCKs represent the most established concepts, yet they are currently concentrated to phase 1/2 trials, with only few phase 2 trials ongoing. In the case of suicide switches, despite the highest number of completed studies, the switches only have been activated in rare cases, precluding conclusions regarding their clinical benefit or optimal formats. Other multifunctional designs are largely restricted to phase 1 trials, with limited reporting also precluding meaningful conclusions on translational progress as of the time of this review. Of note, a recurring translation challenge for several multifunctional CAR T cells is the lack of predictive preclinical models and assays to adequately evaluate safety liabilities such as CRS and immunogenicity. This gap complicates both regulatory assessment and early-stage decision-making. Thus, regulatory frameworks need to evolve to define standards for such cases. While in the absence of robust non-clinical models, some early-phase clinical studies may expedite selection of optimal product candidates to later-phase studies using a parent-child approach. Although this approach has been successfully implemented in the United States, it is not readily applicable within the European regulatory framework, highlighting the need for alternative translational pathways. In future, regulatory alignment will be essential to fully realize the potential of these next-generation strategies and to extend the transformative impact of CAR T cell therapy beyond its current indications.

## Materials and methods

Clinical trials dated until September 2025 were collected from the public database Clinicaltrials.gov using the search terms “chimeric antigen receptor” or “CAR T cells.” Search results were compiled into one dataset, with any duplicates being removed. Data sources were collected from the public domain, including the NCI drug database, research articles, patents, conference abstracts, and the official web pages from study sponsors. For further analysis, we excluded studies that were either observational (86 entries); did not consist of a CAR T cell intervention (454 entries); lacked basic information on the CAR T cell product, e.g., target antigen and clinical indication (123 entries); or investigated more than one disease category at a time, e.g., solid cancer, hematological cancer, or autoimmune diseases (36 entries). For chronological assessment, the date on which the study record was first made available on Clinicaltrials.gov was considered. Data analysis and visualization were performed using R (v.4.2.2). The packages used included dplyr (v.1.1.4) and tidyverse (v.2.0.0) for data wrangling, and ComplexUpSet (v.1.3.6) and ggplot2 (v.3.5.1) for visualization.

To highlight antigens used for multitargeting, the top 20 most frequent antigen pairs were selected for network analysis. The network was constructed by treating each antigen as a node and each pairwise antigen combination as an edge. The strength of these connections was determined by the frequency of co-targeting, highlighting the most commonly tested antigen pairs. Results were visualized using tidygraph (v.1.3.1) and ggraph (v.2.2.1).

## Acknowledgments

This research received no funding from any agency in the public, commercial, or non-profit sectors. The article represents individual opinions of the authors and does not necessarily represent the viewpoints of their employers.

## Author contributions

Conceptualization, I.E.Y.O., N.K., D.L., and B.E.; data curation, I.E.Y.O. and N.K.; formal analysis, N.K. and I.E.Y.O.; visualization, N.K. and I.E.Y.O.; writing – original draft, I.E.Y.O. and N.K.; writing – review & editing, I.E.Y.O., N.K., B.E., M.S., and D.L.; supervision, D.L., B.E., and M.S.

## Declaration of interests

I.E.Y.O., N.K., B.E., and D.L. are employees of Miltenyi Biotec. M.S. has received industry support from Amgen, Bristol Myers Squibb (BMS)/Celgene, Gilead/Kite, Johnson & Johnson, Miltenyi Biotec, Novartis, Roche, Seattle Genetics, and Takeda; serves as a consultant/advisor for AbbVie, Crossbow, Debiopharm, Gilead/Kite, Interius, Johnson & Johnson, Molecular Partners, Novartis, and Otsuka; and serves on the speakers’ bureau at Amgen, BMS/Celgene, Gilead/Kite, Miltenyi Biotec, Novartis, Roche, and Takeda.
